# A high-density consensus linkage map of white lupin highlights synteny with narrow-leafed lupin and provides markers tagging key agronomic traits

**DOI:** 10.1038/s41598-017-15625-w

**Published:** 2017-11-10

**Authors:** Michał Książkiewicz, Nelson Nazzicari, Hua’an Yang, Matthew N. Nelson, Daniel Renshaw, Sandra Rychel, Barbara Ferrari, Maria Carelli, Magdalena Tomaszewska, Stanisław Stawiński, Barbara Naganowska, Bogdan Wolko, Paolo Annicchiarico

**Affiliations:** 1 Institute of Plant Genetics Polish Academy of Sciences, Strzeszyńska 34, 60-479 Poznań, Poland; 2 CREA-FLC, Council for Agricultural Research and Economics, Research Centre for Fodder Crops and Dairy Production, Viale Piacenza 29, 26900 Lodi, Italy; 3 Department of Agriculture and Food Western Australia, 3 Baron-Hay Court, South Perth, 6151 Australia; 4 Natural Capital and Plant Health, Royal Botanic Gardens Kew, Wakehurst Place, Ardingly, West Sussex RH17 6TN UK; 5 UWA Institute of Agriculture, The University of Western Australia, 35 Stirling Highway, Perth, WA 6009 Australia; 6 Plant Breeding Smolice Ltd., Przebędowo 1, Murowana Goślina, 62-095 Poland

## Abstract

White lupin (*Lupinus albus* L.) is a valuable source of seed protein, carbohydrates and oil, but requires genetic improvement to attain its agronomic potential. This study aimed to (i) develop a new high-density consensus linkage map based on new, transcriptome-anchored markers; (ii) map four important agronomic traits, namely, vernalization requirement, seed alkaloid content, and resistance to anthracnose and Phomopsis stem blight; and, (iii) define regions of synteny between the *L. albus* and narrow-leafed lupin (*L. angustifolius* L.) genomes. Mapping of white lupin quantitative trait loci (QTLs) revealed polygenic control of vernalization responsiveness and anthracnose resistance, as well as a single locus regulating seed alkaloid content. We found high sequence collinearity between white and narrow-leafed lupin genomes. Interestingly, the white lupin QTLs did not correspond to previously mapped narrow-leafed lupin loci conferring vernalization independence, anthracnose resistance, low alkaloids and Phomopsis stem blight resistance, highlighting different genetic control of these traits. Our suite of allele-sequenced and PCR validated markers tagging these QTLs is immediately applicable for marker-assisted selection in white lupin breeding. The consensus map constitutes a platform for synteny-based gene cloning approaches and can support the forthcoming white lupin genome sequencing efforts.

## Introduction

White lupin (*Lupinus albus* L., WL) is a legume crop cultivated in ancient Greece and Egypt more than three thousand years ago^1^. Cultivars being developed currently are appreciated as a valuable source of protein (38–42% in seeds)^2^, while having moderate seed content of oil (10–13%) with excellent food quality^3^. These attributes make this species valuable for human food and animal feed^4^. Lupins have positive impact on soil fertility through symbiotic nitrogen fixation and efficient mobilization of soil phosphorus, and are extremely beneficial in crop rotations^5^. Elite germplasm resources with strengthened cold tolerance, determinate growth habit and dwarf architecture have been developed^6–8^. Moreover, untapped landraces with higher yielding ability than current cultivars await thorough exploitation in breeding programmes^9^. However, despite such a long domestication history and considerable opportunities for crop improvement, WL breeders have to struggle with various challenges hampering worldwide cultivation of the species that include, *inter alia*, the exploitation of optimal vernalization requirement for floral initiation and the identification of genetic resistance to devastating diseases, such as anthracnose and Phomopsis stem blight^10–13^. Also, selected varieties have to ensure seed alkaloid content below 200 mg/kg for food utilization and below 500 mg/kg for feed use^14^.

Wild WL requires a period of low temperature to promote flowering, a process known as vernalization. This phenomenon, common in cool season legumes, prevents flowering during winter, when the conditions are too harsh for successful reproduction^15^. Onset of flowering has crucial importance for plant adaptation to specific cropping regions^9^. While late flowering via high vernalization requirement proved essential for expanding autumn sowing in oceanic and subcontinental-climate regions of Europe^16^, intermediate flowering via moderate vernalization proved necessary in autumn-sown environments of southern Europe^17^. However, vernalization is undesirable where WL is a spring-sown crop in cool temperate climates such as in central Europe, because it considerably delays (or even prevents) the crop flowering. Likewise, lack of vernalization requirement is a useful trait for the autumn-sown crop in mild-winter, Mediterranean areas^18^. As global surface temperatures continue to rise, the weather window available for plants to fulfill vernalization requirements is narrowing^19,20^. Therefore, varieties possessing reduced or zero vernalization requirement are likely to become more widespread. One source of early flowering is the recessive locus, *brevis*, which was identified in Kiev Mutant and Ultra varieties in the 1960’s^1^.

Anthracnose of lupins is caused by the pathogenic fungus *Colletotrichum lupini* (Bondar) Nirenberg, Feiler & Hagedorn^21^. WL is generally very susceptible to anthracnose. This disease was first reported in Brazil in 1912, and in 1939 reached the USA^22^. In Europe, anthracnose was first observed in France (1982), and during outbreak of 1995-1997 it wiped out lupin fields from Belarus to the United Kingdom^23^. Despite numerous anthracnose assays, only one WL source of resistance, located in mountainous regions of Ethiopia, has been identified. Three most resistant accessions, namely P27174, P27175, and P27178, were collected in one district and are probably related^10^.

Phomopsis stem blight is caused by the pathogenic fungus *Diaporthe toxica* Williamson, whose anamorph was previously classified as *Phomopsis leptostromiformis* (Kühn) Bubák^24^. This disease was first observed on lupins in Germany, in 1880^25^. The fungus produces mycotoxins, phomopsins A and B, which cause lupinosis, a serious disease of sheep grazing contaminated stubble^26^. Several WL lines with moderate resistance were identified, including two Ethiopian accessions (P28507 and P27174)^27^.

As with many legume crops^28^, the rate of genetic improvement of WL has been slow. Combining early flowering and anthracnose resistance traits in WL took decades of traditional breeding and finally yielded in the release of just two cultivars carrying an incremental improvement of anthracnose resistance^10,29^. Modern breeding tools are required to accelerate the rate of genetic improvement. Molecular genomic resources of WL include a transcriptome assembly^30^, two mapping populations with associated low-density linkage maps^11,31,32^ and sequence tagged site (STS) markers linked to low alkaloid content (PauperM1) and anthracnose resistance (WANR1, WANR2 and WANR3)^4,33^. The main reference recombinant inbred line (RIL) mapping population was developed from the cross Kiev Mutant × P27174. This population segregates for many agronomic traits, including time to flowering, alkaloid content and profile, plant height, pod shape, restricted branching, and resistance to anthracnose and Phomopsis stem blight^11^, most of which have been localized on the WL linkage map^11,31,34^. However, quantitative trait loci (QTL) mapping efforts have so far been limited by low marker density (one marker per 10.8 cM^11^ and 4.6 cM^31^).

Next generation sequencing techniques can generate large numbers of molecular markers at low cost by sequencing single nucleotide polymorphism (SNP) sites in a fraction of the genome. A key advantage of genotyping-by-sequencing (GBS) is a cost reduction of about 40–60% relative to array-based genotyping (albeit with larger proportion of missing data)^35^. For example, GBS in rice generated over 20000 SNP markers that aligned to the reference genome^36^. In pioneer applications of GBS to grain or forage legumes, the number of SNP markers ranged from a few to several thousand depending on the species, the sequencing effort and the missing data threshold^37,38^.

Generation of high-density linkage map of WL, saturated with STS markers, would open possibilities for genome-wide association studies. Such a tool would be later harnessed to identify molecular background underlying major agronomic and physiological traits, as was demonstrated for vernalization responsiveness of narrow-leafed lupin (*Lupinus angustifolius* L., NLL)^39–42^. The development of a set of sequence-defined markers, tightly linked to these traits, would enable implementation of marker-assisted selection in breeding programs, as was achieved in the sister species NLL for early flowering^43^, anthracnose^44–46^ and Phomopsis stem blight^47–49^.

The objectives of this study were (1) to construct a new consensus linkage map of WL integrating those previously published^11,31^ with a rich set of newly developed, sequence-defined markers anchored to the species transcriptome; (2) to provide high-resolution QTL assay of anthracnose resistance, vernalization responsiveness and low alkaloid content, and (3) to investigate synteny between WL and its sister lupin crop, NLL. Besides its important contribution to knowledge on the WL structural and functional genomics, this study aimed to address the emerging demands of breeding community by providing markers tagging QTLs of great agronomic importance.

## Results and Discussion

### Genotyping-by-sequencing (GBS) provided a high-density set of markers

The sequencing of the reduced representation library was performed on four repeated Illumina HiSeq. 2000 lanes, resulting in an average of 1.99 million of reads per RIL sample (from 353341 to 4633907 reads). Appropriate quality datasets were obtained for 189 RILs. Barcode-based demultiplexing assigned 96.81% of obtained sequencing data to plant samples. SNP calling yielded 4830 markers. Following on, markers were divided into three groups based on the segregation distortion Chi-square test: equal segregation, 3405 markers (P > 0.05); moderately distorted, 136 markers (0.001 < P < 0.05); very distorted, 58 markers (1E-07 < P < 0.001); unacceptably distorted, 1231 markers (P < 1E-07). Of 3599 accepted markers, 3481 (96.7%) had unambiguous allelic phases as determined from parental controls. The allelic phases of 116 of the remaining 118 markers were determined through mapping both phases of these markers relative to the unambiguous markers. The remaining two markers were excluded from further analysis, while 3597 markers were retained. The average observed heterozygote frequency for RIL lines for the set of 3597 markers was 0.38%. Marker sequences are provided in Supplementary Table S1. These markers increased 8-fold the total number available for WL, confirming the capability of GBS to expand considerably the marker suite applicable for genetic studies. Likewise, GBS provided thousands of SNP markers in another cool-season grain legume species without a reference genome sequence, pea^50^. Genotyping data for 465 published WL markers^4,11,31,33^ were verified by preliminary linkage mapping. Marker phase was revealed to be inverted for 76 markers (75 “Pms” markers and Lup270). Segregation data, Chi-square values and allele frequencies for all markers are provided in Supplementary Table S2.

### Consensus WL linkage map carries 3669 sequence-defined markers

Some 4062 markers (3597 developed in this study, and 465 from two previous maps) were subjected to linkage mapping. 196 RILs were assayed, including 189 RILs genotyped in this study and 7 RILs missing new sequencing data but having genotype and phenotype scores from previous surveys. A total of 166 complete multipoint mapping runs were performed (from 5 to 10 per linkage group) to establish the final version of the map. Some 99.7% of the analyzed markers were mapped (4050), using 97.9% as “skeleton” (i.e., contributing to calculation of recombination frequencies), and 2.1% as “attached” (with position assigned approximately in relation to adjacent skeleton markers). The standard deviation (SD) of skeleton relative marker position (in Kosambi cM), calculated for the last five Joinmap outputs, ranged from 0 to 7.27, with an average value of 1.08 (Supplementary Table S3). Markers from two previously published maps^11,31^ had higher average SD of relative position than those developed in this study (1.34 vs 1.05). Such a difference may have resulted from the different number of lines genotyped (94 vs 189), as well as from differences in the reliability of genotyping method applied. Twelve previously published markers failed to match any linkage groups. STS markers constituted 90.59% of the consensus linkage map; 98.04% of which were generated in this study (Table 1). As marker grouping was performed using high independence LOD threshold (11.0) and was followed by several rounds of linkage group reconstructions with the removal of markers showing the nearest neighbour fit/stress values higher than 3.0 cM, only well-fitting markers were incorporated to linkage map. An average distance to markers with distorted segregation was 0.80 (Joinmap 4.1^51^) or 1.61 cM (DistortedMap 1.0^52^), whereas an average distance to markers with normal segregation was 0.73 (Joinmap 4.1) or 1.54 cM (DistortedMap). The influence of distorted markers on the linkage map length was revealed to be negligible, therefore we chose to retain such markers in the consensus map.

**Table 1:** Summary statistics of the consensus linkage map of white lupin.

**Linkage group**	**Length (cM, Joinmap)**	**Loci**	**Hetero-zygotes (%)**	**Skeleton markers**	**Attached markers**	**Markers with distorted segregation (P < 0.05)**	**Sequenced markers (STS)**
ALB01	171.07	135	0.40	281	13	13	253
ALB02	134.33	99	0.56	215	5	11	186
ALB03	122.02	100	0.34	209	3	10	201
ALB04	128.75	91	0.30	200	6	10	182
ALB05	110.61	87	0.40	201	3	15	185
ALB06	132.11	86	0.39	171	2	8	159
ALB07	117.89	82	0.27	171	1	7	163
ALB08	124.57	82	0.42	164	4	11	161
ALB09	119.13	83	0.30	159	7	11	140
ALB10	119.12	79	0.34	164	0	7	154
ALB11	109.32	64	0.33	154	2	7	145
ALB12	101.61	75	0.25	145	5	7	139
ALB13	101.31	76	0.22	142	5	6	119
ALB14	123.75	83	0.25	143	4	11	121
ALB15	124.53	72	0.27	141	6	8	130
ALB16	121.59	73	0.31	146	1	8	141
ALB17	109.47	68	0.34	137	4	7	123
ALB18	155.86	82	0.32	139	1	5	132
ALB19	107.37	68	0.46	139	3	5	135
ALB20	107.87	67	0.36	137	1	14	126
ALB21	118.49	68	0.28	133	1	7	128
ALB22	89.88	66	0.30	127	4	4	122
ALB23	93.34	67	0.35	127	2	23	116
ALB24	84.63	61	0.38	111	1	8	104
ALB25	121.41	60	0.22	110	0	5	104
Total	2950.03	1974	0.34	3966	84	228	3669

**Figure 1:**
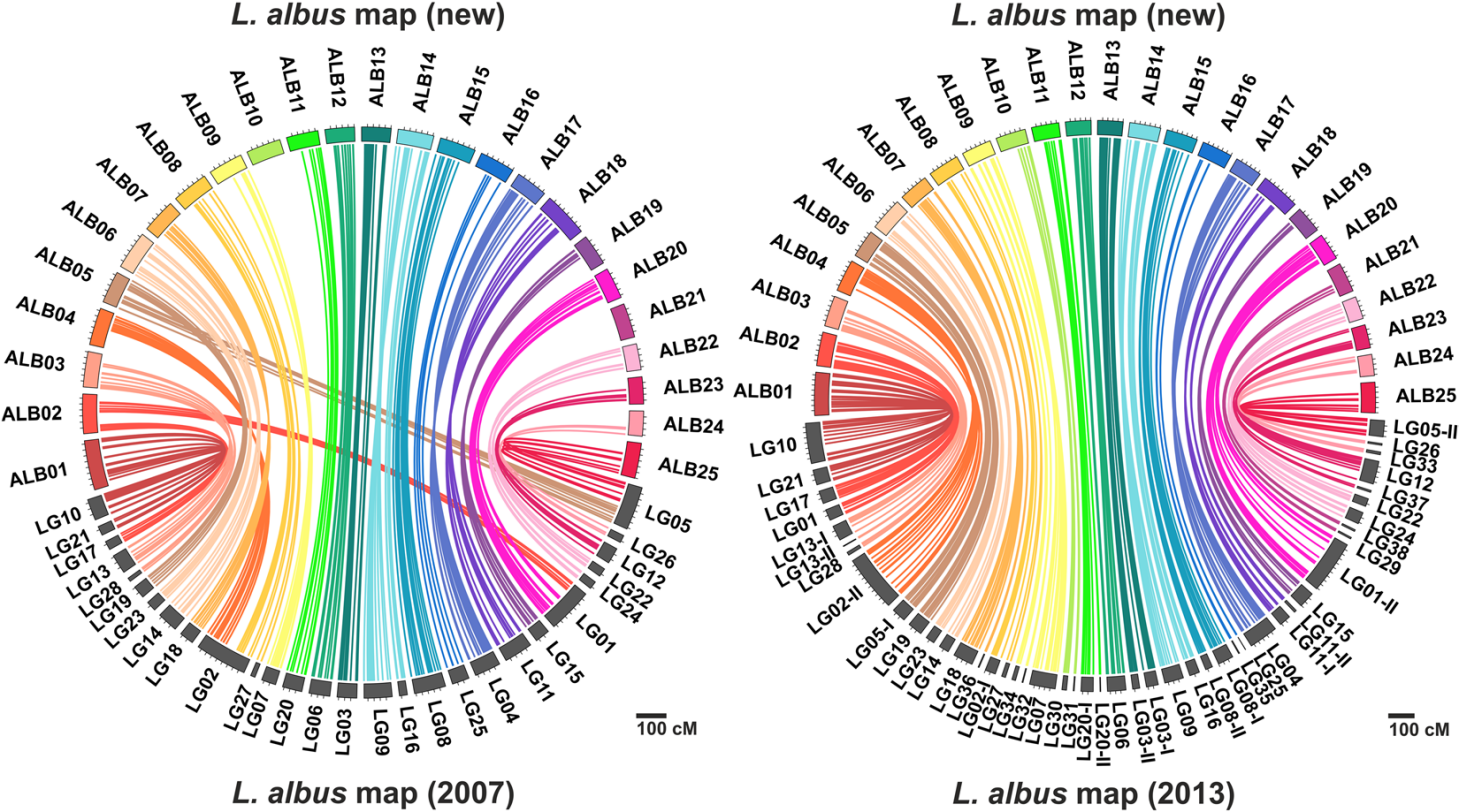
Links between the consensus white lupin linkage map and two previously published maps^11,31^. Ribbons match common markers used for map integration. Linkage groups are drawn according to cM scale.

In total, 25 linkage groups were constructed (ALB01 - ALB25), consistent with the 25 chromosome pairs reported for this species^53^. All linkage groups from two previous maps (hereafter, ‘2007’ and ‘2013’) were matched (Fig. 1). No linkage group split was observed for the 2013 map^31^ but three splits were observed relative to the 2007 map^11^. Merging occurred for 21 linkage groups from the 2013 map (Supplementary Table S4) and for 5 from the 2007 map (Fig. 1). Twelve markers which failed to localize on the new map originated from the middle sections of linkage groups of these maps. Therefore, we could conclude that both maps were fully integrated into the consensus.

### High level of collinearity exists between WL and NLL genomes

**Figure 2:**
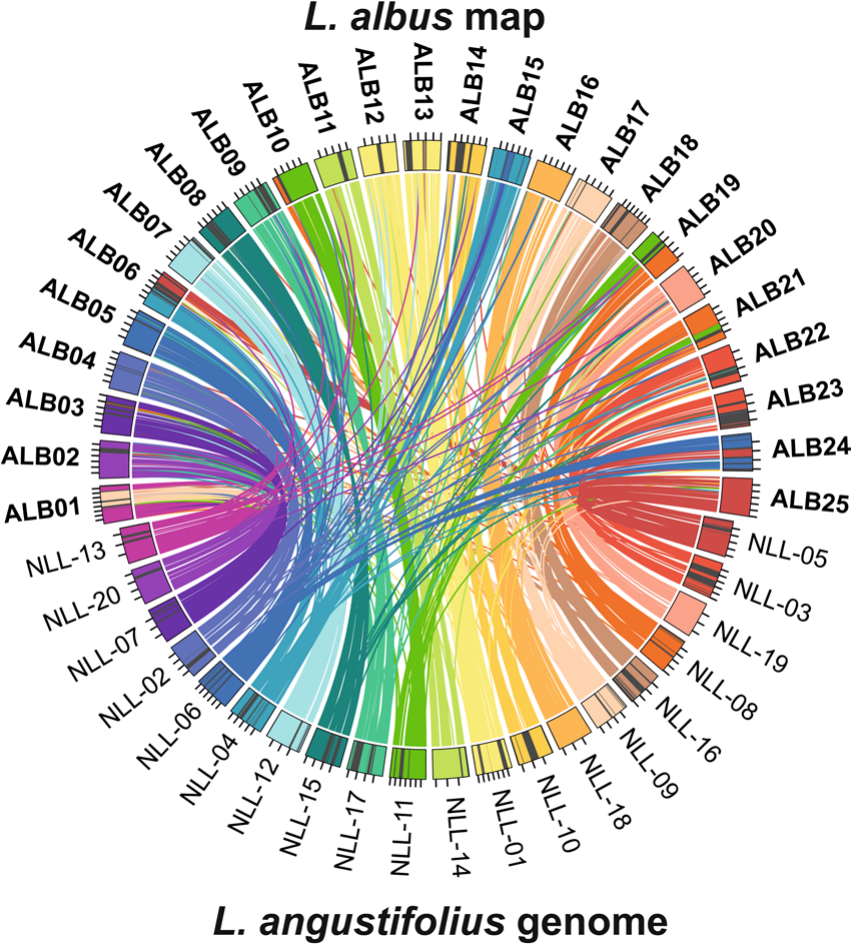
Collinearity links matching white (ALB01 – ALB25) linkage groups and narrow-leafed lupin (NLL-01 – NLL-20) pseudochromosomes. Ribbons symbolize homologous links identified by DNA sequence similarity. Chromosomes and linkage groups are not drawn to scale.

Kiev Mutant and P27174 transcriptome assemblies were developed, to generate longer sequence anchors for comparative analysis. Sequencing bulked RNA samples extracted from young leaves, floral buds and developing pods provided 45.9 million reads for Kiev Mutant, and 69.7 million for P27174. Trinity assembly yielded 110799 contigs for Kiev Mutant and 109726 for P27174, with an average length of 715 bp and 805 bp, respectively. RNAseq datasets are available at the NCBI short read archive (BioProject PRJNA380248). High stringency alignment of Kiev Mutant, P27174 and reference LAGI01^30^ transcriptome assemblies to the WL linkage map provided anchors for 55.7% of sequence-defined markers. WL transcriptome alignment data and annotations from the *L. angustifolius* gene index^41^ and *A. thaliana* TAIR10 assembly are provided in Supplementary Table S5. These transcriptome-anchored sequences (2043), together with the remaining unmatched sequenced markers (1626), were mapped to the NLL genome, yielding non-redundant alignments for 3230 WL markers (Supplementary Tables S6 and S7). In total, 58 large (longer than 10 cM) syntenic blocks were identified, constituting 78.8% of total WL linkage group length and unambiguously matching 73.9% of total NLL pseudochromosome assembly length (347.8 of 470.4 Mb). The average length of large WL syntenic blocks was 40.0 cM, whereas that of corresponding NLL genome segment was 6.0 Mb. Besides large blocks, 36 smaller segments of sequence collinearity were identified. Total lengths of all WL vs NLL syntenic blocks were estimated as 2472.4 cM vs 381.5 Mb, respectively. Thirteen NLL blocks were larger than 10 Mb. Several WL linkage groups revealed strikingly high similarity to single NLL chromosomes, demonstrated by very long segments of collinearity and high total coverage, i.e. ALB02 to NLL-20, ALB04 to NLL-02, ALB07 to NLL-12, ALB11 to NLL-14, ALB14 to NLL-10, ALB16 to NLL-18, ALB17 to NLL-09, ALB20 to NLL-19, and ALB25 to NLL-05 (Fig. 2). Some NLL chromosomes revealed fragmented macrosyntenic anchors to three WL linkage groups, such as NLL-04, NLL-08 and NLL-11. However, no WL linkage group showed large collinearity links to more than two NLL chromosomes (Fig. 2, Supplementary Table S8). The synteny pattern may indicate that WL is more similar to the ancestral form than NLL. Given that there appeared to be a whole genome triplication event around 24.6 mya^41^, it seems likely that NLL has undergone more chromosome fusions than WL. This would be analogous to *A. thaliana* whose 5 chromosome pairs appears to have fused from 8 ancestral chromosome pairs, hence the value of the more un-rearranged genome configuration of *A. lyrata* in comparative genomics^54,55^. This would increase the value of WL as a model, especially once its genome becomes available.

### Vernalization responsiveness in WL is under polygenic control

**Figure 3:**
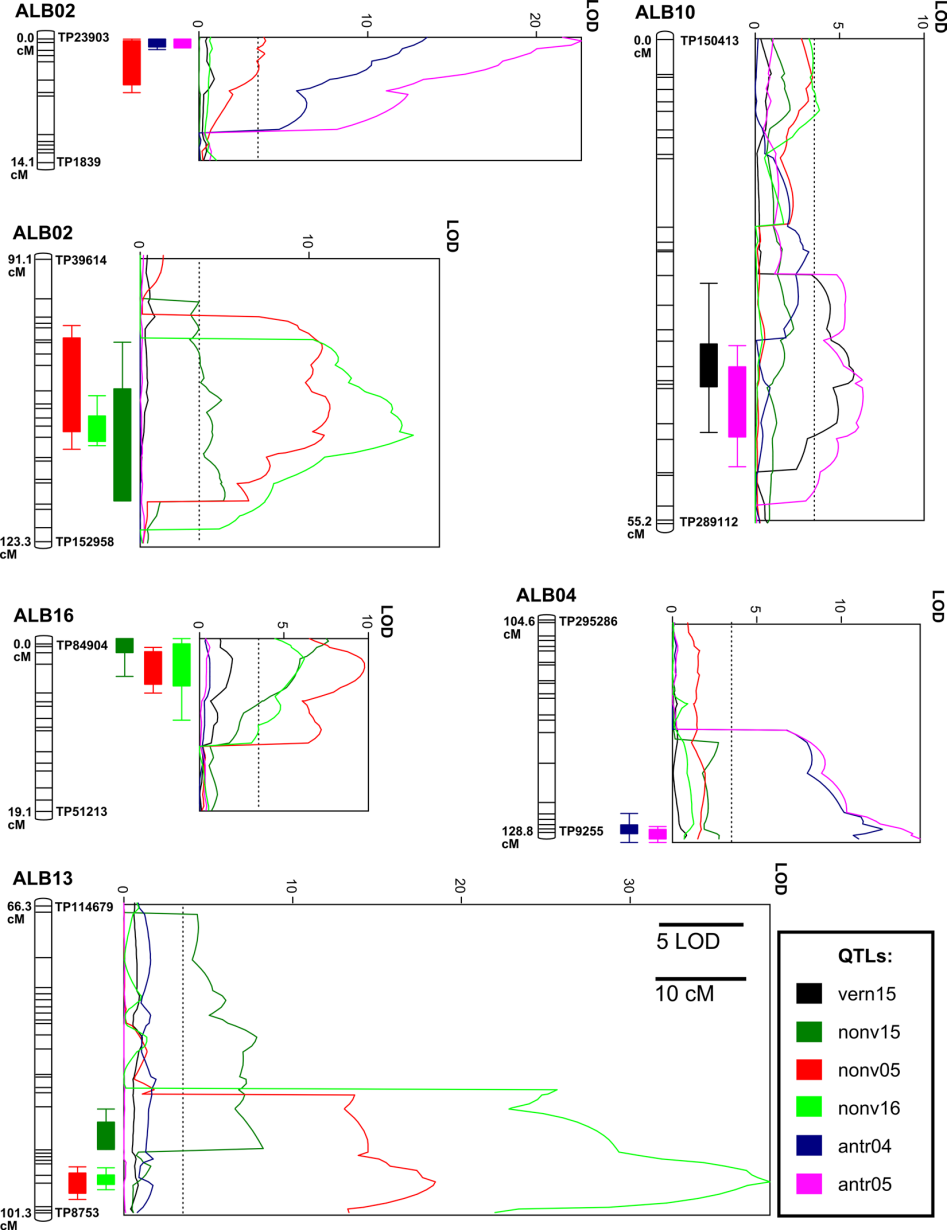
Major QTLs for time to flowering and anthracnose resistance. Linear plots show LOD values (threshold 3.5), rectangles LOD-based QTL ranges (LOD 2.0 and 1.0 below the maximum value) whereas bar graphs visualize corresponding linkage group fragments. Linkage groups are drawn to scale. Colors corresponds to QTL assays, time to flowering (black, vern15, vernalized plants in Przebędowo; green, nonv15, non-vernalized plants in Przebędowo; light green, nonv16, non-vernalized plants in Poznań; red, nonv05, non-vernalized plants in Perth) and anthracnose resistance (pink, antr04, Perth 2004; blue, antr05, Perth 2005).

QTL mapping was performed using three methods: interval mapping, IM (MapQTL 6^56^), composite interval mapping with background markers, CIM (Windows QTL Cartographer 2.5) and genome-wide composite interval mapping, GCIM (mrMLM package^57^). Four QTLs were identified for vernalization responsiveness in the Polish field experiment by IM, three by CIM and three by GCIM (Table 2). Four QTLs were found in the Polish greenhouse experiment. All methods provided similar localizations and additive effect estimates for three major QTLs. Four to five QTLs were found in the Australian field experiment by these methods. Loci of three QTLs were consistent across all experiments (nonv05, nonv16 and nonv15 traits on linkage groups ALB02, ALB13 and ALB16; Fig. 3), suggesting the involvement of the same set regulatory factors (genes or heritable epigenetic marks). The explained phenotypic variance for each QTL ranged from 6.5% to 41.9%. The positions of two major QTLs (on linkage groups ALB02 and ALB13) correspond to coordinates of those previously published^11,31^. Phenotype scores used for QTL mapping are provided in Supplementary Table S9 and QTL mapping results are provided in Supplementary Table S10. Kiev Mutant has been reported to carry a single recessive early flowering gene, *brevis* ^1^. However, instead of a 1:1 segregation ratio expected of a single gene in a RIL population, continuous variation for flowering time was observed in RILs here and in other crosses involving Kiev Mutant^10^. In the absence of vernalization, we found the most influential QTL located on ALB13 and controlling up to 41.9% of phenotypic variation (Fig. 3). Taking into consideration the IM/CIM LOD distribution across the linkage group ALB13 and the resolution of interval mapping methods, the presence of a pair of quite closely linked QTLs might be hypothesized. It is possible that the previously announced gene *brevis* may relate to this gene (or genes). However, it is clear from the occurrence of multiple QTLs for flowering time that early flowering in Kiev Mutant is under polygenic control, which supports previous observations of low frequencies of early flowering plants in F_2_ and downstream progenies of Kiev Mutant × late lines crosses (~4%)^10,11^. Thus, while the *brevis* allele may be influential, it comprises a relatively modest proportion of the early flowering trait in Kiev Mutant. In addition to *brevis*, other recessive genes conferring early flowering phenotype (*floridus*, *festinus*, and *contractus*) were described in WL germplasm; however, their relation to Kiev Mutant or P27174 genes remains unknown^58^. Recent investigation of a world WL collection identified several accessions with considerably reduced time to flowering, including Start (cultivar) and P28283 (French breeding line)^29,59^. Crossing of Start and P28283 with late flowering lines revealed that early flowering in these lines is controlled by two complementary dominant genes *Ef1* and *Ef2*, different than those of Kiev Mutant and Ultra^59^.

**Table 2:** Flowering time QTLs detected in a recombinant inbred line population of white lupin with (+) and without (−) vernalization treatment.

**QTL**	**Linkage group**	**Interval mapping (MapQTL6)**				**Composite interval mapping (WinQTL Cartographer)**				**Genome-wide composite interval mapping (mrMLM)**			
		**Position (cM)**	**LOD**	**Additive effect**	**PVE**	**Position (cM)**	**LOD**	**Additive effect**	**PVE**	**Position (cM)**	**LOD**	**Additive effect**	**PVE**
**vernalization−, Przebędowo, 2015**													
nonv15_1	ALB02	107.6	4.34	1.07	11.1	121.1	5.02	0.93	8.5	117.8	5.32	0.41	9.9
nonv15_2	ALB13	81.0	6.43	1.29	16.1	—	—	—	—	84.1	7.79	0.49	13.9
nonv15_3	ALB13	94.1	6.20	1.28	15.8	93.7	8.25	1.18	13.7	—	—	—	—
nonv15_4	ALB16	0.0	5.03	1.21	13.5	0.31	7.64	1.14	12.40	3.9	5.58	0.42	10.5
**vernalization−, Perth, 2004/2005**													
nonv05_1	ALB02	104.7	6.70	0.31	16.6	104.7	13.32	0.49	14.7	111.4	7.78	0.28	14.1
nonv05_2	ALB13	82.0	10.92	0.38	24.9	—	—	—	—	—	—	—	—
nonv05_3	ALB13	97.0	11.45	0.38	25.8	97.5	18.13	0.47	20.4	97.4	12.33	0.38	24.8
nonv05_4	ALB16	2.6	3.18a	0.22	8.3	3.30	9.78	0.25	9.7	0.6	3.73	0.19	6.5
nonv05_5	ALB02	4.7	2.65^a^	0.20	6.9	0.4	3.95	0.15	3.7	4.7	3.84	0.20	7.2
**vernalization−, Poznań, 2016**													
nonv16_1	ALB02	114.0	6.87	2.19	16.0	114.1	16.18	2.10	13.9	114.5	14.46	2.22	15.4
nonv16_2	ALB13	81.8	16.25	3.19	34.5	—	—	—	—	—	—	—	—
nonv16_3	ALB13	97.2	18.42	3.39	38.0	97.5	38.29	3.64	41.9	97.4	28.48	3.44	37.0
nonv16_4	ALB16	5.39	2.38^a^	1.37	6.1	2.3	6.21	1.27	5.1	4.6	4.37	1.17	4.3
nonv16_5	ALB24	—	—	—	—	1.0	5.05	1.15	4.0	1.4	3.89	1.08	3.6
**vernalization** + **, Przebędowo, 2015**													
vern15_1	ALB10	36.5	3.06^a^	1.28	8.4	38.3	5.85	1.62	12.97	—	—	—	—

^a^LOD value below genome-wide permutation test threshold but above linkage group thresholds.

PVE - proportion of phenotypic variance explained by QTL.

Besides QTLs overlapping in both Polish and Australian phenotyping experiments, one additional minor effect QTL in the Australian experiment was identified by all methods (nonv05_5). Such an observation may result from differences between environmental conditions between these two locations. Przebędowo experienced average minimum and maximum temperature about 3 °C higher than Perth during phenotyping surveys, accompanied total precipitation lower by 32–42% (Supplementary Table S11). White lupins are considered as facultative long day plants, accelerating flowering under long photoperiod both in controlled environment and field conditions^60^. Theoretical day length based on geographic latitude was approximately 5-6 hours longer in Przebędowo than in Perth. Thus, Polish conditions were more favorable for plants requiring long day to initiate flowering than those recorded in Perth. Days-to-flowering trait may be responsive to plant density. It was reported for *A. thaliana* that 8-fold difference in plant density between experiments (200 vs 1600 plants per square meter) affected a part of flowering time QTLs^61^. Here, plants were grown in similar densities (~67 plants/m^2^ in Perth and ~100 plants/m^2^) in Przebędowo, therefore this issue should have negligible influence on early flowering QTL mapping.

The Polish experiment based on vernalized plants revealed the existence of only one weak QTL confirmed by IM and CIM methods and located in different region than the QTLs found without the vernalization treatment. Such an observation strongly suggests that the major genes influencing induction of flowering in non-vernalized experiments were those involved in the vernalization pathway. As vernalization is the main factor accelerating flowering of wild lupins in general^12^, the presence of non-overlapping QTLs between vernalized and non-vernalized plants is an expected outcome. For all QTLs, tightly linked, sequence-defined markers were identified in the study.

### NLL genome regions syntenic to those carrying WL vernalization responsiveness QTLs encode homologs of known flowering regulatory genes

**Figure 4:**
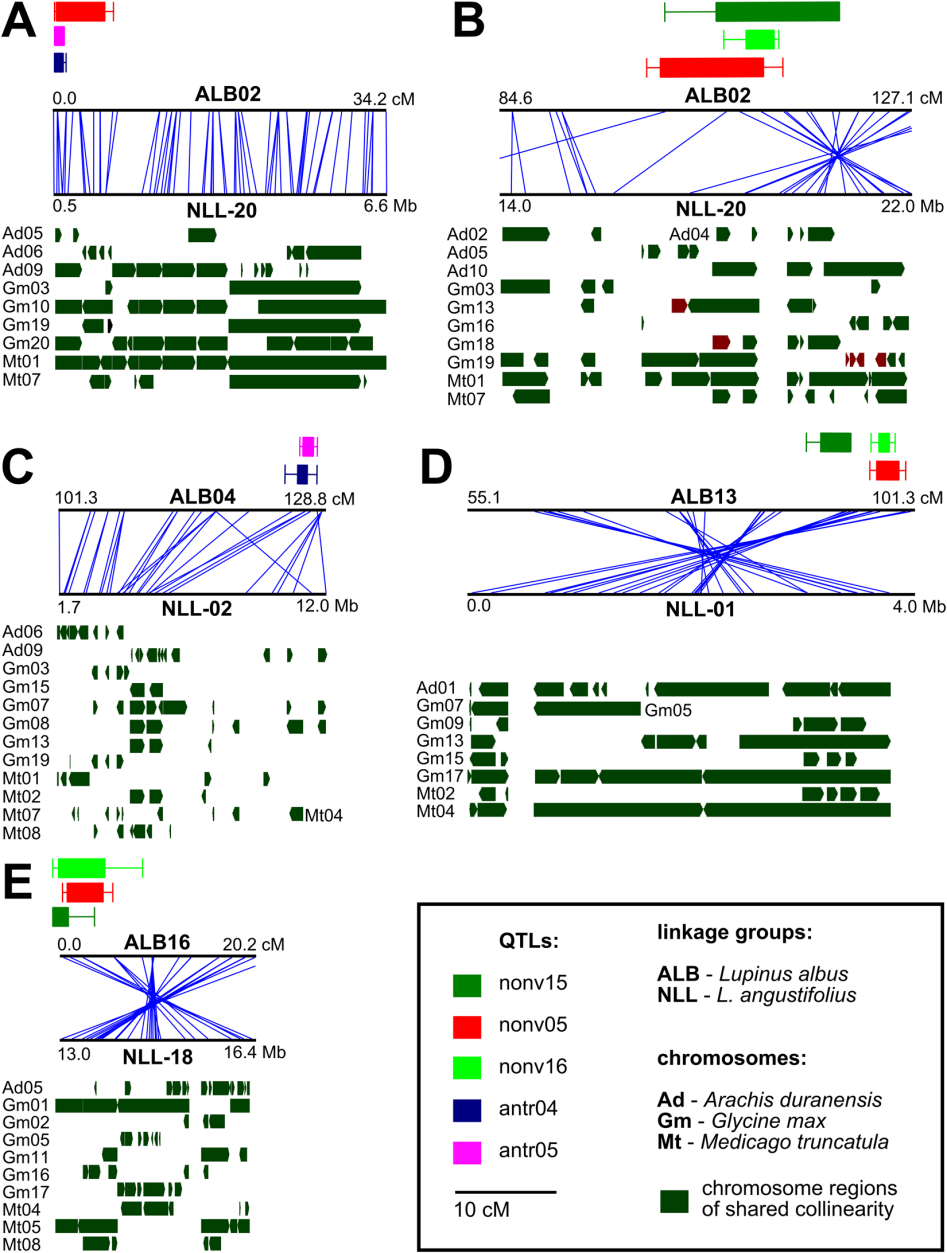
Macrosyntenic links to the narrow-leafed lupin (NLL) genome identified in regions carrying white lupin (WL) QTLs. Five diagrams (**A** to **E**) show different regions of WL genome. Each diagram is composed of three sections - QTL rectangles (up), NLL vs WL synteny graphs (middle) and legume collinearity bars (down). QTL rectangle color corresponds to QTL assay as follows: time to flowering (green, nonv15, non-vernalized plants in Przebędowo; light green, nonv16, non-vernalized plants in Poznań; red, nonv05, non-vernalized plants in Perth) and anthracnose resistance (pink, antr04, Perth 2004; blue, antr05, Perth 2005). Synteny graphs are composed of two horizontal lines; the upper presents WL linkage group fragment (cM coordinates), while the lower shows the corresponding NLL chromosome region (Mb localizations). Legume collinearity bars placed below NLL chromosomes shows syntenic regions of *Arachis duranensis* (Ad), *Glycine max* (Gm) and *Medicago truncatula* (Mt) chromosomes are shown as myrtle green horizontal bars.

All QTLs identified for vernalization responsiveness of WL have been located in regions showing shared collinearity to the NLL genome. Moreover, corresponding NLL regions revealed also preserved collinearity to reference legume genomes: *Arachis duranensis*, *Glycine max* and *Medicago truncatula* (Fig. 4). Analysis of syntenic regions revealed that none of the WL early flowering QTLs match the region of chromosome NLL-10 recently shown to contain the vernalization independence *Ku* locus^39,42^ (Table 2, Supplementary Table S8). The NLL *Ku* locus encodes a *FLOWERING LOCUS T* homolog, *LanFTc1*. NLL vernalization independence is thought to be conferred by 1.4 kb insertion/deletion in the *LanFTc1* promoter region. Based on synteny analysis, WL vernalization independence appears not to be controlled by the same *FT* homologue as in NLL^42^.

Insight into NLL genome regions syntenic to WL QTLs enabled us to identify some candidate genes, namely: another *FT* homolog (Lup021189) for nonv15_1/nonv16_1/nonv05_1, *SQUAMOSA PROMOTER BINDING–LIKE* (Lup018483) for nonv15_2/nonv16_2/nonv05_2, *F-BOX SKIP1* (Lup009500) for nonv15_3/nonv16_3/nonv05_3, and *DNAJ* (Lup007587) for nonv15_4/nonv16_4/nonv05_4. All these genes could hypothetically contribute to flowering initiation^62–65^. Further work is required to verify these candidates.

It has been hypothesized that vernalization pathways evolved in the temperate Cenozoic era when global cooling occurred and differed between plant families which were already separated at that time^66–68^. Whole-genome triplication, which could have provided novel copies of regulatory genes, putatively occurred in the genistoid lineage ~24.6 million years ago (Mya)^41^. Considerable decreases in global temperature are estimated to have happened near the Eocene/Oligocene boundary (~36 Mya), in the Miocene (~15 Mya) and near the end of the Pliocene (~2.5 Mya)^69^. Therefore, vernalization pathways in WL and NLL might have evolved independently to each other, but the divergence time between these two species has not yet been estimated.

### Anthracnose resistance of WL is controlled by two major QTLs

Two major QTLs (confirmed by all three methods) were revealed for the 2004 anthracnose resistance experiment and four for the 2005 experiment (Table 3; Fig. 3). Tightly linked flanking markers were identified for all these QTLs. Two major QTLs explaining jointly more than 40% of phenotypic variance were localized at highly overlapping positions in both experiments, indicating the involvement of two heritable factors located on linkage groups ALB02 and ALB04. These two QTLs correspond to two loci identified previously in the 2007 map on linkage groups LG4 and LG17, respectively, which previously were found to explain 31% and 26% of trait variance^11^. Due to high fragmentation of two preceding maps, no linkage between anthracnose resistance and vernalization responsiveness was observed^11,31^. However, improvement of linkage map resulted in merging several linkage groups, including those carrying major QTLs for early flowering (ALB02, ~4.7 cM and ~104.7 cM) and anthracnose resistance (ALB02, 0.4 cM). Combining these traits by conventional breeding proved difficult and crop improvement was not achieved at the desired level with the use of Kiev Mutant as early flowering donor and Ethiopian landraces as the source of anthracnose resistance^10,59^. PCR and microarray-based screening of a core collection of WL germplasm confirmed that Ethiopian accessions represent a unique genepool^70^. Replacement of Kiev Mutant by French germplasm in WL breeding resulted in milestone achievement in WL crop improvement, as no linkage between these two traits was observed^29^. The present study provides a genetic explanation for this phenomenon, as well as delivers molecular markers to track both Kiev Mutant early flowering alleles and P27174 anthracnose resistance genes in breeding progenies.

**Table 3:** Anthracnose resistance QTLs detected in a recombinant inbred line population of white lupin.

**QTL**	**Linkage group**	**Interval mapping(MapQTL6)**				**Composite interval mapping(WinQTL Cartographer)**				**Genome-wide composite interval mapping(mrMLM)**			
		**Position (cM)**	**LOD**	**Additive effect**	**PVE**	**Position (cM)**	**LOD**	**Additive effect**	**PVE**	**Position (cM)**	**LOD**	**Additive effect**	**PVE**
**Perth, 2004**													
antr04_1	ALB02	0.0	9.03	−0.56	27.5	0.0	13.52	−0.59	28.1	3.3	10.15	−0.54	25.1
antr04_2	ALB04	125.8	5.21	−0.42	16.3	127.3	12.43	−0.51	23.4	126.0	7.38	−0.42	15.2
antr04_3	ALB23	65.8	4.35	0.30	15.6	—	—	—	—	—	—	—	—
**Perth, 2005**													
antr05_1	ALB02	0.4	10.34	−0.43	24.5	0.4	22.66	−0.45	26.7	3.3	16.30	−0.44	23.5
antr05_2	ALB04	124.2	4.46	−0.29	11.4	128.4	14.67	−0.35	15.5	126.0	9.26	−0.31	11.7
antr05_3	ALB10	21.9	3.37^a^	−0.26	9.3	40.79	6.39	−0.22	6.8	40.8	5.39	−0.23	6.6
antr05_4	ALB13	—	—	—	—	47.1	6.02	−0.22	6.3	44.5	4.74	−0.21	5.5
antr05_5	ALB17	46.1	2.80^a^	−0.25	8.7	31.5	10.08	−0.28	9.9	32.1	6.11	−0.25	7.4

^a^LOD value below genome-wide permutation test threshold but above linkage group thresholds.

PVE - proportion of phenotypic variance explained by QTL.

Polygenic control of anthracnose resistance is uncommon. Resistance to *Colletotrichum* spp. in legumes is usually conferred by single dominant genes, recognizing narrow range of pathogen races and following the gene-for-gene model^71^. *RCT1* in *Medicago truncatula* is a single dominant gene acting directly against *C. trifolii* race 1^72^. The genome of *Phaseolus vulgaris* contains numerous monogenic dominant loci providing race-specific resistance to *C. lindemuthianum*, localized in six chromosomes^73^. Several single dominant genes control also resistance against anthracnose in NLL germplasm, namely *Lanr1* in cv. Tanjil, *AnMan* in cv. Mandelup and *LanrBo* in line Bo7212^44,46,74^. The localization of WL anthracnose resistance QTLs in blocks of conserved sequence collinearity enabled us to address the positions of these NLL loci (Fig. 4). None of WL anthracnose resistance QTLs matched NLL genome regions carrying *Lanr1* or *Anman* genes, providing high and moderate levels of resistance to *C. lupini* in NLL^41,46,49,75^. QTL antr05_3 may hypothetically correspond to multi-strain *C. lupini* resistance *LanrBo* locus in NLL, located in the middle of linkage group NLL-11^74^. However, intervals between anchor markers flanking *LanrBo* in Bo7212-derived linkage map are too large to draw any firm conclusions. Analysis of the NLL genome region showing collinearity to WL QTLs provided two candidate genes for antr04_1/antr05_1 locus, encoding LRR protein kinase (Lup026475 and Lup026461). No candidate for antr04_2/antr05_2 was found.

### Major low alkaloid genes in WL and NLL are different

Alkaloid content was observed as a binary trait (bitter or sweet) and segregated in a 1:1 ratio (Chi-square P = 0.64) consistent with Mendelian expectations for a single locus. It was mapped by linkage mapping in the same way as the molecular markers. Alkaloid locus was localized with high confidence LOD value of 37 in the linkage group ALB18 (Table 4, Supplementary Table S3). We also observed no unexpected double crossovers in relation to adjacent molecular markers, which demonstrates the unambiguous position of this Mendelian trait locus. One co-segregating and two tightly linked flanking markers were identified for this locus, which refers to *pauper* gene, widely introduced to modern cultivars^4^. White lupin reveals quite complex pattern of minor alkaloids but a clear influence of domestication on the major alkaloid component (lupanine) was observed^76^ Besides *pauper*, several other natural recessive low alkaloid mutations in WL germplasm were described, including *exiguus*, *mitis*, *nutricius* and *reductus* genes. *Exiguus* and *nutricius* were also bred into certain cultivars^77,78^. Three natural low alkaloid genes were early identified in NLL, namely *depressus*, *esculentus* and *iucundus* ^79^, however only *iucundus* was exploited for NLL breeding^1^. NLL genome region encoding *iucundus* gene (NLL-07) revealed conserved synteny to WL linkage group ALB03, whereas *pauper* gene was localized on ALB18. This finding is in line with recent quantitative and qualitative seed alkaloid content assays, which revealed the partly different pattern of alkaloid compound variation in these species and, occasionally, a different major component influencing total alkaloid content of these species, i.e., lupanine in WL, and lupanine or 13-hydroxylupanine in NLL^14,76,80^. Moreover, it is hypothesized that *pauper* and *iucundus* genes differ by function because these species differ by lysine profiles among wild and sweet accessions^81^.

**Table 4:** Localization of alkaloid content locus (this study) and approximate positions of published QTLs for *D. toxica* resistance (based on flanking markers) in white lupin.

**QTL**	**Linkage group**	**LOD**	**PVE**	**Flanking sequenced marker loci**	**Narrow-leafed lupin syntenic loci (Mb)**
**Alkaloids**					
alkaloid	ALB18	37.0	—	66.6–69.4(3)^b^	NLL-16, ~18.7;NLL-19, ~15.3^a^
**Phomopsis, experiment 1** ^34^					
phtj1_2	ALB13	9.3^c^	36.9^c^	75.6–79.7(11)	NLL-01, ~3.0–2.1
phtj1_4	ALB12	2.1	10.0	82.4–93.5(11)	NLL-01, ~18.7–19.4
phtj1_5	ALB01	6.4	27.1	76.9-93.5(22)	NLL-09, ~8.4–10.4
phtj1_6	ALB23	4.3	19.4	67.7-69.7(3)	n/a
phtj1_7	ALB02	1.8	8.3	0.4–6.5(25)	NLL-20, ~0.5–1.5
**Phomopsis, experiment 2** ^34^					
phtj2_1	ALB13	2.5	11.5	95.3–95.7(5)	NLL-01, ~1.3–0.8
phtj2_3	ALB13	7.1	29.8	55.1–67.0(17)	NLL-16, ~17.5–17.0;NLL-01, ~3.8–3.0^a^
phtj2_5	ALB01	6.7	28.2	76.9–93.5(22)	NLL-09, ~8.4–10.4
phtj2_8	ALB08	1.7	8.0	105.8–120.7(26)	NLL-15, ~19.1–20.8

^a^Some QTLs were located on the border of two syntenic blocks and for those QTLs both positions are provided.

^b^Position (in cM) and the number of flanking markers (provided in parentheses).

^c^Values provided by Cowley *et al*.^34^.

PVE - proportion of phenotypic variance explained by QTL.

The WL *pauper* locus matches the *L. angustifolius* genome region encoding sequences annotated as HXXXD-type acyl-transferases (Lup021583 and Lup021586). Lup021586 gene reveals 100% nucleotide identity to *LaAT* gene (AB581532.1). *LaAT* belongs to BAHD acyl-CoA dependent acyltransferase superfamily and was demonstrated to be highly expressed in quinolizidine alkaloid-producing plants but undetectable in the sweet ones^82^. As the quinolizidine alkaloid biosynthesis pathway is only partially elucidated, no candidate gene for *pauper* has been identified so far^81^. Verification of hypothetical involvement of WL *LaAT* homolog in low alkaloid profile requires extensive studies involving differential transcriptome and metabolome profiling of mapping population parents, representative subset of RILs as well as selected sweet and bitter accessions.

### WL Phomopsis stem blight resistance QTLs do not match NLL *Phr1* and *PhtjR* genes

Localization of QTLs carrying hypothetical genes conferring increased level of WL *D. toxica* resistance was tentatively estimated by incorporating data on published flanking markers^34^. Numerous skeleton markers were mapped within the ranges of Phomopsis stem blight resistance QTLs, but the resolution of the previous map^31^ was too low to provide narrow flanking DNA landmarks directly targeting QTL loci. Therefore, the presented positions of *D. toxica* resistance loci (Table 4) should be considered as preliminary, tentative evaluations requiring further assessment. Several donors of Phomopsis stem blight resistance exist in WL collection, including wild accessions from Turkey (P27664), Syria (P27840, P28096) and Ethiopia (P28507, P27174) as well as cultivars from Australia (Andromeda, WALAB2008), France (Lublanc), Portugal (Madeira), Ukraine (Borki), and Germany (Ultra)^27,34,83^. Association mapping of 94 WL lines collected from 26 countries resulted in selection of numerous microarray-based DArT markers showing significant association with Phomopsis stem blight resistance, however, not a single marker was sequenced^83^. Contrary to WL, *D. toxica* resistance in NLL is controlled by single genes. There are at least three independent NLL donors of *D. toxica* resistance: very resistant line 75 A:258 (gene *Phr1*), moderately tolerant cultivar Merrit (gene *Phr2*) and resistant cultivar Tanjil (gene *PhtjR*)^84,85^. The approximate positions of *Phr1* and *PhtjR* genes in NLL genome assembly were recently determined^41,48,49^. Interestingly, no collinearity link between any of WL Phomopsis stem blight resistance QTLs and NLL regions carrying *Phr1* and *PhtjR* genes was identified in this study (Table 4).

### Validation of SNP genotypes for PCR-based genotyping

Attempts were undertaken to implement early flowering and anthracnose resistance QTL-targeting markers in plant breeding using PCR amplification and sequencing (Table 5). Moreover, to supplement this analysis Kiev Mutant and P27174 transcriptome assemblies were aligned to confirm particular SNPs. Amplicons were obtained for all 17 markers analyzed. Expected Kiev Mutant and P27174 sequences were revealed for all PCR product except one (TP253114). Positive verification was obtained for markers matching all major QTLs, namely antr04_1/antr05_1 and antr04_2/antr05_2 for anthracnose resistance, as well as nonv05_1/nonv15_1/nonv16_1, nonv05_2/nonv15_2/nonv16_2, nonv05_3/nonv15_3/nonv16_3 and nonv05_4/nonv15_4/nonv16_4 for early flowering. Markers for some minor QTLs were also investigated and positively verified, including antr04_3, antr05_3 and antr05_5 for anthracnose resistance as well as nonv05_5 and vern15_1 for early flowering. Marker sequences were deposited in GenBank under accession numbers: MF630883-MF630916.

**Table 5:** Results of candidate QTL markers verification by PCR amplification and Sanger sequencing.

**Target QTLs**	**Marker**	**Group**	**Locus cM**	**Template sequence**	**Sanger sequencing verification**
antr04_1, antr05_1, nonv05_5	TP222136	ALB02	0.38	LAGI01_42695	+
antr04_2, antr05_2	TP61122	ALB04	113.03	LAGI01_32421	+
antr04_2, antr05_2	TP26007	ALB04	127.3	LAGI01_59788	+
antr04_3	TP233608	ALB23	63.93	LAGI01_17609	+
antr05_3	TP251482	ALB10	23.95	LAGI01_13427	+
antr05_5	TP37582	ALB17	51.13	LAGI01_20131	+
nonv05_1, nonv15_1, nonv16_1	TP12311	ALB02	100.6	LAGI01_71758	+^b^
nonv05_1, nonv15_1, nonv16_1	TP56963	ALB02	107.61	LAGI01_2487	+
nonv05_2, nonv15_2, nonv16_2	TP114679	ALB13	66.3	LAGI01_31630	+
nonv05_2, nonv15_2, nonv16_2	TP263618	ALB13	77.81	LAGI01_47039	+^c^
nonv05_3, nonv15_3, nonv16_3,	TP345457	ALB13	94.06	LAGI01_13490	+
nonv05_3, nonv15_3, nonv16_3,	TP288595	ALB13	97.85	LAGI01_30547	+
nonv05_4, nonv15_4, nonv16_4	TP2488	ALB16	0.30	LAGI01_6453	+^c^
nonv05_4, nonv15_4, nonv16_4	TP253114	ALB16	0.97	LAGI01_68341	−
vern15_1, antr05_3	TP252015	ALB10	30.29	LAGI01_22063	+
vern15_1, antr05_3	TP1928	ALB10	39.31	P_comp40124_c0_seq. 1	+

^+^Positively verified by PCR amplification and Sanger sequencing.

^b^Positively verified by PCR amplification and Sanger sequencing but only partial amplicon sequence was obtained.

^c^Positively verified by Kiev Mutant and P27174 transcriptome sequencing.

^−^Negatively verified by PCR amplification and Sanger sequencing - amplification of other sequence.

## Conclusions

GBS was confirmed as a breakthrough technique for inexpensive genotyping of WL, a species without a reference genome sequence, making available nearly 3,600 markers with normal segregation. The WL consensus map that we generated constitutes a platform that can enable synteny-based gene cloning approaches and can support future WL genome sequencing efforts. A second important finding of our study is the occurrence of high collinearity between WL and NLL genomes. Using this new understanding of genome collinearity, we showed that four key traits are controlled by different regions in WL and NLL. A third, key finding of this study is the different control of early flowering and anthracnose resistance in *L. albus* compared with reference *L. angustifolius* accessions on the grounds of different number of genes involved (several QTLs vs single genes), different inheritance patterns (recessive vs dominant) and putatively different genes or homologs involved (visualized by lack of matching of corresponding trait positions between these genomes despite well-conserved synteny). This has considerable implications for our ability to exploit the genetic information made available by a close, genome-sequenced species such as NLL for the purpose of WL improvement, and reinforces the need for genome sequencing of WL rather than relying on NLL as a proxy genome. Finally, this study served the WL breeding community by making available a batch of allele-sequenced markers tightly linked to QTLs for vernalization independence, anthracnose resistance, low alkaloid profile and Phomopsis stem blight resistance, whose application can be devised immediately for marker-assisted selection of the crop.

## Methods

### Plant material

Genetic and QTL mapping was performed using the reference Kiev Mutant × P27174 F_8_ RIL population (n = 196), provided by the Department of Agriculture and Food Western Australia (South Perth, Australia). This population was derived from a single plant cross between a vernalization-responsive, anthracnose resistant Ethiopian landrace P27174 and a vernalization-independent, anthracnose susceptible Ukrainian cultivar Kiev Mutant^4^. F_2_ seeds harvested from a single F_1_ plant were advanced to F_8_ RILs by single seed descent.

### Vernalization responsiveness

RILs and founding parental lines were assayed in the field experiment with and without pre-sowing vernalization at Plant Breeding Smolice Ltd. station located in Przebędowo, Poland (52°35′N 17°01′E) in 2015. Vernalization was carried out by placing imbibed seeds for 21 days at 5 °C in darkness on moist filter paper in Petri dishes. Non-vernalized control plants were sown five days before the end of vernalization experiment and kept at ~21 °C to maintain a similar thermal time^16^. Both treatments were hand sown (5 cm between plants in row and 20 cm between rows) and grown under ambient long day photoperiod (14–16 h) with recorded temperature and precipitation (Supplementary Table S11). Time to flowering was recorded daily, when the first fully opened flower was observed. Data constituted two series, “nonv15” carrying non-vernalized plants (181 RILs), and “vern15” - vernalized (184 RILs). Greenhouse experiment without pre-sowing vernalization was performed at the Institute of Plant Genetics of the Polish Academy of Sciences in Poznań (52°26′N 16°54′E) in 2016 under ambient long day photoperiod (14–16 h), providing “nonv16” dataset (192 RILs).

A second assay was performed in a screen house in 2003, and in field nurseries in 2004 and 2005 in Perth, Western Australia (31°59′S 115°53′E), without vernalization. Temperature and precipitation data were derived from the Perth meteorological station (31°56′S 115°58′E) (Supplementary Table S11). Each line was grown in 2 m rows (5 cm between plants in row and 30 cm between rows). Flowering was recorded when 50% plants in the row had one or more open flower on the main stem inflorescence^11,31^. The scale from 1 (the earliest) to 6 (the last) was used. Due to high level of similarity (average variation between these two sets was 0.29), observations from Australian surveys were averaged (arithmetic mean of all 2004 and 2005 repeats) and used as one input, “nonv05” (190 RILs). Different number of RILs with phenotyping data results from two factors: seed availability and plant survival during experiments.

### Anthracnose resistance

Anthracnose resistance profiling was performed in two experiments (Perth, years 2004 and 2005), involving three independent replicates. RILs and parental lines were grown in 2 meter rows. Each line had three replicates. Inoculum seedlings (Kiev Mutant) were maintained in a glasshouse at 20 °C. Fourteen days after sowing, plants were sprayed with spore suspension (10^5^/mL) of *C. lupini* isolate 96A4 (IMI375715) and incubated for 24 h. Ten days after inoculation, infected seedlings were transplanted into disease nurseries. Disease assessment was done when plants had begun to senesce. The percentage of plants with anthracnose symptoms on the main stem or lateral branches were recorded three times as continuous variables employing a scale from 1 (highly resistant) to 5 (extremely susceptible) (Thomas and Sweetingham 2004, Yang *et al*. 2010). Only partial results were used before due to linkage map constraints^11,33^. Here, the whole dataset was exploited. Resistance scores from three repetitions were averaged for each year and implemented as series “antr04” (152 RILs) and “antr05” (191 RILs).

### Alkaloid content

Alkaloid content in 189 RILs and two parental lines was scored using two techniques (Dragendorff paper test and UV fluorescence) and encoded as a binary trait (bitter or sweet). Methods and partial results were presented elsewhere^4^. Here, we incorporated the whole dataset developed in that study.

### Transcriptome profiling of Kiev Mutant and P27174 lines

Seeds of parental lines were vernalized as described above. RNA was extracted with TRIzol reagents (Invitrogen) using the method optimized for lupins^40^ from young leaves, floral buds and developing pods and then pooled in equal measure. The quantity and quality of total RNA were measured using Qubit (Invitrogen) and BioAnalyzer (Agilent) assays. HiSeq. 2000 sequencing of 100PE cDNA libraries was performed by Macrogen Korea Inc. Transcriptome assemblies were generated by Macrogen using the Trinity assembler^86^.

### Development of sequence-based marker dataset

Seeds of 191 RILs and parental lines were sown in a glasshouse under ambient long day photoperiod at the Institute of Plant Genetics, Polish Academy of Sciences (Poznan, Poland; 52°26’N, 16°54’E) in 2013. Leaf tissue was collected from single three week old plants. Plant DNA was isolated using DNeasy Plant Mini Kit (Qiagen) and quantified with a Quant-iT^TM^ PicoGreen® dsDNA assay kit (Life Technologies). Library preparation protocol^35^ was modified. Each DNA sample (100 ng) was digested with *Ape*KI (New England Biolabs) and ligated to unique barcode and common adapters. Equal volumes of ligated products were pooled and purified with NucleoSpin^®^ Gel and PCR Clean-up (Macherey-Nagel). Template DNA (50 ng) was mixed with two primers and KAPA Library Amplification Readymix (KAPA Biosystems). Amplification steps were as follows: 5 min at 72 °C, 30 s at 98 °C, and 10 cycles with 10 s at 98 °C, 30 s at 65 °C and 30 s at 72 °C. Sequencing was performed on four Illumina HiSeq. 2000 lanes (University of Texas at Austin, USA).

Evaluation of quality scores was done using fastqc (http://www.bioinformatics.babraham.ac.uk/projects/fastqc/). Demultiplexing was performed by fastq-multx^87^. SNP calling was executed in Tassel UNEAK^88,89^ (parameters in Supplementary Table S12). Genotype scores were obtained in ad-hoc likelihood based SNP caller using the estimated sequencing error rate, 0.01; the minimum number of reads per locus, 2; and the minimum ratio of called genotype likelihood over all candidate likelihoods (a measure of the SNP calls reliability), 0.796 (the minimum value to accept a 2-reads locus). A missing value per marker threshold of 0.6 was applied. As attempting to impute missing data points in this condition would be very prone to errors^90^, no imputation was forced. Genotype codes were assigned as follows: a, P27174; b, Kiev Mutant; h, heterozygote; -, no data. A Chi-squared test of segregation distortion from expected for RIL_8_ a/h/b ratio (49.61/0.78/49.61) was applied. Markers with P value below 1E-07 were discarded from further analysis.

### Construction of the consensus linkage map

Segregation data from two previous maps^11,31^ were imported to Map Manager QTXb20^91^ and manually inspected. To match all three datasets and phenotyping surveys, a consensus list of 196 RILs was established. Corrected segregation files, together with those developed in this study, were imported to JoinMap 4.1^51^. After grouping under independence LOD of 11.0 multipoint mapping was performed (parameters in Supplementary Table S12). Markers with nearest neighbour fit and nearest neighbour stress values higher than 3.0 cM were removed and mapping was repeated as many times as required to maintain these values below 3.0. Four additional mapping runs were performed using the established set of markers to calculate standard deviation of relative marker position. Markers removed during recombination frequency optimization procedure, were localized in the linkage map using Map Manager QTXb20 (map function Kosambi, search linkage criterion P = 1e-3) and classified as “attached”. Their approximate positions were calculated by linear interpolation of adjacent skeleton markers. Additionally, DistortedMap 1.0^52^ was used to analyze genetic distances between distorted markers.

### Comparative mapping to NLL genome

Marker sequences were aligned to WL transcriptome assemblies, both the reference (LAGI01)^30^ and those developed in this study (Kiev Mutant and P27174), allowing one nucleotide mismatch per marker and one lacking nucleotide per alignment. One best hit per marker was allowed. A Fasta file with marker sequences replaced by assigned transcripts (if applicable) was used for comparative mapping by BLAST^92^ to the NLL genome assembly^41^ (Supplementary Table S12). Based on the results of comparative mapping to NLL genome, minor alterations in marker order within tight microsyntenic clusters were applied to establish a final version of consensus map.

### Quantitative trait loci mapping

Interval mapping^56^ was performed using MapQTL 6 (Kyazma, Wageningen, Nederlands) (Supplementary Table S12). In order to determine the significance threshold of the LOD score under the null-hypothesis (no QTL present), a permutation test (1000×) was performed. Based on the results of this test (Supplementary Table S13), the LOD threshold of 3.5 was used for QTL determination. QTLs were localized at positions with the highest LOD values. QTL boundaries were assigned according to LOD values: outer, 2.0 below maximum; inner, 1.0 below maximum. Composite interval mapping was performed in Windows QTL Cartographer V2.5 (North Carolina State University, Raleigh, USA) using 20 background control markers window size 10 cM and walk speed 0.5 cM. Genome-wide composite interval mapping was executed in the mrMLM package^57^. SNP genotypes of candidate markers linked to the QTLs were validated by using Sanger sequencing of amplicons for further PCR-based genotyping. Primer sequences and annealing temperatures used for PCR during SNP validation were provided in the Supplementary Table S14.

### Visualization

Linkage groups were drawn in MapChart^93^. Sequence collinearity blocks were visualized using Genome Synteny Viewer^94^, Circos^95^ and Legume Information System^96^.

### Data availability

All data generated during this study are included in this published article, its Supplementary Information files and in public repository (NCBI short read archive PRJNA380248 and accessions MF630883-MF630916).

## Electronic supplementary material

Supplementary Table S1

Supplementary Table S2

Supplementary Table S3

Supplementary Table S4

Supplementary Table S5

Supplementary Table S6

Supplementary Table S7

Supplementary Table S8

Supplementary Table S9

Supplementary Table S10

Supplementary Table S11

Supplementary Table S12

Supplementary Table S13

Supplementary Table S14
