# Supplementary material for: A high-density consensus linkage map of white lupin highlights synteny with narrow-leafed lupin and provides markers tagging key agronomic traits
Source: Sci Rep. 2017 Nov 10;7:15335. doi: 10.1038/s41598-017-15625-w (PMC5681670; doi:10.1038/s41598-017-15625-w)
Supplement: Supplementary file 12 — Supplementary Table S12 [file 41598_2017_15625_MOESM12_ESM.doc]

**A high-density consensus linkage map of white lupin highlights synteny with narrow-leafed lupin and provides markers tagging key agronomic traits**

**Michał Książkiewicz, Nelson Nazzicari, Hua’an Yang, Matthew N. Nelson, Daniel Renshaw, Sandra Rychel, Barbara Ferrari, Maria Carelli, Magdalena Tomaszewska, Stanisław Stawiński, Barbara Naganowska, Bogdan Wolko, Paolo Annicchiarico**

**Supplementary Table S12. Parameters used for white lupin SNP calling, linkage map construction, comparative mapping and QTL determination.**

SNP calling (Tassel UNEAK) parameters:

- FastqToTagCountPlugin, -e ApeKI -s 2000000000;
- MergeTaxaTagCountPlugin, -c 5 -m 200000000 -x 100000000;
- TagCountToTagPairPlugin, -e 0.03;
- UmapInfoToHapMapPlugin, -mnMAF 0.01 -mxMAF 0.5 -mnC 0 -mxC 1.

JoinMap 4.1 consensus map construction parameters:

- mapping algorithm, maximum likelihood;
- spatial sampling thresholds, 0.1, 0.05, 0.03, 0.02, 0.01;
- map optimization rounds per sample, 5;
- chain length, 2500;
- initial acceptance probability, 0.25;
- cooling control parameter, 0.00025;
- chains without improvement limit, 50000;
- length of burn-in chain, 50000;
- Monte Carlo expectation-maximization cycles, 10;
- chain length per cycle, 1000;
- sampling period for recombination frequency matrix samples, 5.

BLAST parameters used for comparative mapping to the narrow-leafed lupin genome:

- -task blastn-short,
- -evalue 1e-5,
- -max target sequences 3,
- max hsps 1.

Quantitative trait loci interval mapping parameters:

- algorithm, mixture model;
- test statistic, LOD;
- mapping step size, 0.1;
- maximum no. of iterations, 200;
- functional tolerance, 1.0e-08;
- population type, RI8;
- dominance fitted, no;
- QTL genotype probability approximation, no;
- maximum no. of neighboring markers used, 10.
